# Appetitive and aversive outcome associations modulate exogenous cueing

**DOI:** 10.3758/s13414-016-1107-6

**Published:** 2016-05-04

**Authors:** Berno Bucker, Jan Theeuwes

**Affiliations:** Department of Experimental and Applied Psychology, Vrije Universiteit Amsterdam, Van der Boechorststraat 1, 1081 BT Amsterdam, The Netherlands

**Keywords:** Visual attention, Exogenous cueing, Attentional capture, Reward, Punishment

## Abstract

In two experiments, we utilized an exogenous cueing task in which different-colored abrupt-onset cues were associated with an appetitive (gain of 10 cents), aversive (loss of 5 cents), or neutral (no gain or loss) outcome. Reward delivery did not depend on performance, but instead the specific exogenous cues were always followed by their corresponding outcome in a classical-conditioning–like manner. Compared to neutral cues and independent of cue–target delay, the results of Experiment 1 showed that appetitive cues strengthened attentional capture, whereas aversive cues reduced attentional capture. The data revealed that both appetitive and aversive cues initially facilitated responding at the validly cued location. At the long cue–target delays, however, this facilitation effect at the validly cued location remained present for gain-associated cues while it reversed for loss-associated cues. The results of Experiment 2 confirmed these findings by showing that both neutral and aversive cues initially facilitated responding at the cued location and that, at long cue–target delays, aversive cues elicited stronger reorienting away from the cued location as compared to neutral cues. Together these findings indicate that all abrupt-onset cues initially capture attention independent of their outcome association. Yet, if time passes, attention remains lingering at the location of gain-associated cues, whereas attention is released and reoriented away from the location of loss-associated cues. Altogether, we show that associating the color of an abrupt-onset cue with an appetitive or aversive outcome can modulate attentional deployment following exogenous cueing.

For efficient everyday behavior we constantly have to select information from our visually rich world. Attention is the mechanism that selectively enhances and suppresses parts of the incoming information so that certain objects or locations in the visual field can be processed in more detail at the cost of others. Traditionally, a distinction has been made between endogenous and exogenous factors driving visual selective attention (Posner, 1980). Endogenous factors are often referred to as “top down” and guide attention in a voluntary, controlled, and goal-driven manner. Exogenous factors are often referred to as “bottom up” and guide attention in an involuntary, automatic, and stimulus-driven manner. The probability that a specific part of the incoming visual information is selected depends on the interplay between the “exogenous” physical features of the environment (i.e., salience) and the “endogenous” control settings of the observer.

Exogenous and endogenous attentional selectivity can be inferred from performance in spatial cueing tasks (Jonides, 1981; Posner & Cohen, 1984). To examine the allocation of endogenous spatial attention, typically, a central cue (e.g., an arrow) indicates the location where the target is likely to appear (i.e., on more than 50 % of the trials). As the endogenous cue predicts the target location in the majority of trials, this gives observers an incentive to use the cue and voluntarily direct their attention toward the location of the cue before the target is presented (Jonides, 1981). Typically, performance improves in terms of reaction time and/or accuracy when the target is presented at the cued compared to the uncued location. To examine the allocation of exogenous spatial attention, typically, an abrupt onset or luminance transient presented in the periphery is used that summons attention to its location. Unlike endogenous cues, exogenous cues do not predict the target location (i.e., 50 % cue–target validity), providing no incentive for the observer to attend to the cued location. However, it has been argued that exogenous cues capture attention toward their location in an automatic, bottom-up manner, even when this runs counter to the intentions of the observer (Jonides, 1981; Posner & Cohen, 1984; Remington, Johnston, & Yantis, 1992; Theeuwes, 1991, Yantis & Jonides, 1990).

A striking difference between endogenous and exogenous attention is the time course of their deployment in space. Endogenous attentional orienting develops relatively slowly but can be sustained and lasts for a longer period of time at the attended location, whereas exogenous attentional orienting develops relatively quickly but is transient and disappears rapidly from the attended location (see Egeth & Yantis, 1997). This biphasic pattern of exogenous attention is well described in Posner and Cohen (1984). Participants fixated on a central box while two other boxes were presented in the periphery. During a trial, the outline of one of the peripheral boxes briefly brightened and served as the exogenous cue. At variable cue–target delays, the target was displayed inside one of the boxes, and observers detected its presence by pressing a single key as quickly as possible. The results showed that responses were faster when the cue and target appeared at the same location compared to different locations, but only when the cue–target delay was shorter than 200 ms. At longer cue–target delays, the opposite pattern was observed, with faster responses occurring when the cue and target appeared at different locations. It was argued that the initial capture of attention toward the location of the exogenous cue caused rapid and early facilitation, followed by inhibition of that location and reorienting toward the uncued location. This inhibitory effect due to exogenous spatial orienting was termed “inhibition of return” (IOR; see Klein, 2000). IOR is typically observed only when attention is exogenously captured and does not occur following endogenous attentional orienting (Schreij, Theeuwes, & Olivers, 2010; Theeuwes & Godijn, 2002).

In the present study, we investigated attentional orienting and reorienting following exogenous cueing in two behavioral experiments. More specifically, we examined whether the attentional processes that follow exogenous cueing could be modulated by associating appetitive and aversive outcomes (i.e., gains and losses) with different-colored abrupt-onset cues. A recent body of literature has shown that reward associations can evoke attentional biases that cannot be considered to be driven by exogenous or endogenous factors (see Anderson, 2013, 2015; Awh, Belopolsky, &Theeuwes, 2012; Chelazzi, Perlato, Santandrea, & Della Libera, 2013). Stimuli associated with high reward have been shown to receive attentional priority over equally salient competing stimuli associated with low or no reward (e.g., Bucker, Silvis, Donk, &Theeuwes, 2015; Failing & Theeuwes, 2014; Kiss, Driver, & Eimer, 2009; Krebs, Boehler, Egner, & Woldorff, 2011; Stankevich & Geng, 2015), disregarding an exogenously driven bias based on the physical salience of the stimuli. Furthermore, distractors associated with high reward have been shown to capture attention to a greater extent than distracters associated with low or no reward when observers are searching for targets in a goal-directed manner (e.g., Anderson, Laurent, & Yantis, 2011a, b; Bucker, Belopolsky, & Theeuwes, 2015; Bucker & Theeuwes, 2016; Failing, Nissens, Pearson, Le Pelley, & Theeuwes, 2015; Lee & Shomstein, 2013; Le Pelley, Pearson, Griffiths, & Beesley, 2015). Awh et al. (2012) proposed that rewards belong to a separate class of attentional biases that influence selection by the significance that certain stimuli have gained over time trough experience. They argued that the attentional priority map should be extended beyond the integration of the physical salience of stimuli and the goals of the observer and include “selection history” as a third factor driving visual selective attention.

A clear example of reward associations influencing attentional processing comes from studies showing value-driven attentional capture (see Anderson, 2013). For example, during an initial training phase, Anderson and colleagues (Anderson et al., 2011a, b) had observers search for a red or green target circle among multiple colored circles and discriminate the orientation of a line segment within the target circle. For correct responses observers earned either a high or low reward ,depending on the color-reward contingencies (e.g., green was associated with a high probability of receiving high reward and a low probability of receiving low reward, and red was associated with a low probability of receiving high reward and a high probability of receiving low reward). Then, during a following test phase, observers had to search for an odd-shaped target among several distractors and discriminate the orientation of a line segment within the target shape (i.e., the additional singleton paradigm; Theeuwes, 1992). Rewards could no longer be earned, but one of the distractor shapes could occasionally be presented in the previously high- or low-reward associated color. Even though observers were instructed to ignore color information, the distractor slowed search significantly when it was presented in the previously high-reward associated color compared to the previously low-reward associated color and compared to when neither color was present. This shows that stimuli associated with high reward can capture attention when they are nonsalient and task irrelevant, which suggests that value-driven attentional capture cannot be explained in terms of exogenous or endogenous attention. Very recently, several studies (Bucker & Theeuwes, 2016; Failing et al., 2015; Le Pelley et al., 2015) nuanced this finding by showing that a reward bias like this is not a result of repeated selection of the high-reward associated stimulus in the training phase (i.e., instrumental learning) but rather that the reward bias is established by associative learning about stimulus–reward contingencies (i.e., Pavlovian learning). Together these studies show that reward associated stimuli enjoy high attentional priority and elicit value-driven attentional capture.

As reward-associated stimuli and pure exogenous abrupt-onset cues both capture attention, we questioned whether abrupt-onset cues associated with positive (gain) or negative (loss) outcomes could modulate the deployment of exogenous attention. In two experiments, we utilized a classic Posner cueing task in which different-colored abrupt-onset cues were presented at one of two possible target locations. Crucially, the presentation of specifically colored exogenous cues was associated with an appetitive (gain of 10 cents), aversive (loss of 5 cents), or neutral (no gain or loss) outcome. We specifically focused on the Pavlovian “signal value” of the abrupt-onset cues by making gains and losses independent of behavioral performance. This means that the appetitive and aversive cues certainly predicted the monetary outcome of that trial (i.e., a gain of 10 cents and a loss of 5 cents, respectively). Because monetary losses could not be avoided by giving a fast or accurate response, we were able to examine valence-specific effects of the “signal value” associated with abrupt-onset cues. In Experiment 1, participants were exposed to monetary wins and losses by presenting appetitive, aversive, and neutral abrupt-onset cues, intermixed. In Experiment 2, aversive and neutral abrupt-onset cues were presented in a blocked manner to focus on the deployment of exogenous attention following cues predictive of a monetary loss.

We expect that if capture is an all or none phenomenon, and pure exogenous abrupt-onset cues exert the maximum amount of capture possible, then it is unlikely that adding an appetitive or aversive signal to this cue will modulate capture. The pure luminance onset of the signal drives capture, and there is no modulation due to the association with a monetary gain or loss. However, if saliency-driven and value-driven capture share the same underlying mechanism, and their effects are additive within the attentional priority map, one expects that the appetitive and aversive signals may modulate the strength of capture. We hypothesized that an abrupt-onset cue associated with a monetary gain might exert a stronger effect than that very same cue when it is not associated with any outcome. However, our expectation of how aversive abrupt-onset cues modulate attention could go in opposite directions. On the one hand, value-driven mechanisms might trigger more attraction toward the location of the aversive cue compared to a neutral cue because a cue leading to a monetary loss (i.e., a negative reward value) might be interpreted as more informative or arousing than a cue that is not associated with a monetary outcome. Indeed, it has been shown that using the additional singleton task, a stimulus associated with an electric shock (i.e., an aversive stimulus) can capture attention more strongly than an equally salient stimulus that is not associated with an electric shock (Schmidt, Belopolsky, & Theeuwes, 2015). On the other hand, valence-driven mechanisms might trigger reorienting away from the location of the aversive cue because a cue associated with a monetary loss might be interpreted as negative or less attractive than a cue that has a neutral outcome.

## Experiment 1

To investigate whether exogenous cueing could be modulated by value, we adapted the classic Posner cueing task. An abrupt-onset cue followed by a short or long cue–target delay preceded target presentation. Participants fixated on the fixation cross at all times and performed a target discrimination task at one of two possible peripheral locations while a distractor stimulus was shown at the other peripheral location. In Experiment 1, we utilized a mixed design in which the presentation of one of three different-colored abrupt-onset cues was associated with an appetitive (+10 cents), an aversive (-5 cents) or a neutral (+0 cents) outcome. We chose asymmetrical loss versus gain values because, as a general rule, the subjective value of losses is larger than the subjective value of gains (see Tversky & Kahneman, 1991).

## Method

### Participants

Thirty-two participants (19 females, 19–33 years of age, mean = 24.5 years, standard deviation = 3.2 years) were recruited, who received €6.00 for participation and could earn €4.40 extra reward if their accuracy was above 75 % correct. All participants reported having normal or corrected-to-normal vision and gave written informed consent before participation. All research was approved by the Vrije Universiteit Faculty of Psychology ethics board and was conducted according to the principles of the Declaration of Helsinki. For counterbalancing reasons, two participants were replaced by others because their accuracy was below chance level in one or more conditions.

### Stimuli and apparatus

All participants were tested in a sound-attenuated, dimly lit room, with their head resting on a chin rest at a viewing distance of 70 cm. A computer with a 3.0 GHz Intel Core processor running OpenSesame (Mathôt, Schreij, & Theeuwes, 2012) generated the stimuli on a 22-in. screen (resolution 1,680 × 1,050, refreshing at 120 Hz). The necessary response data were acquired through the standard keyboard, and auditory stimuli were presented through headphones. All visual stimuli were showed on a black (CIE: *x* = .068, *y* = .567; 0.71 cd/m2) background with a white (CIE: *x* = .255, *y* = .437; 67.11 cd/m2) fixation cross (0.27°) at the center of the screen. At the start of the trial, two gray (CIE: *x* = .298, *y* = .511; 22.12 cd/m2) squared boxes (2.2° × 2.2°) were presented 5° visual degrees left and right of the fixation cross on the horizontal meridian. The exogenous abrupt-onset cue was a brief (150 ms) change of color of the outline of one of the two boxes. The abrupt-onset cue could either be presented in green (CIE: *x* = .293, *y* = .609; 22.17 cd/m2), orange (CIE: *x* = .970, *y* = .466; 22.25 cd/m2), or purple (CIE: *x* = .230, *y* = .195; 22.81 cd/m2). In Experiment 1, these three colors were coupled with an appetitive, aversive, or neutral outcome, and the color–reward contingencies were counterbalanced across participants. A horizontally or vertically gray (CIE: *x* = .298, *y* = .511; 22.12 cd/m2) line of 12 pixels long presented at the center of one of the boxes served as the target stimulus. A similar gray diagonal distracter line appeared at the center of the other box. Reward feedback for correct responses consisted of the written text “+10 ct,” “-5 ct,” or “0 ct” presented 0.50^0^ visual degrees above the fixation cross in the corresponding reward color for appetitive, aversive, or neutral abrupt-onset cues, respectively. Feedback for incorrect and too-slow responses consisted of the same reward feedback above the fixation cross, in addition with the written text “incorrect” or “too slow” presented 0.50^0^ visual degrees underneath the fixation cross. Furthermore, a pure tone (sine of 400 Hz) was played for 100 ms if a response was incorrect and a pure tone (sine of 800 Hz) was played for 100 ms if a response was too slow.

**Fig. 1:**
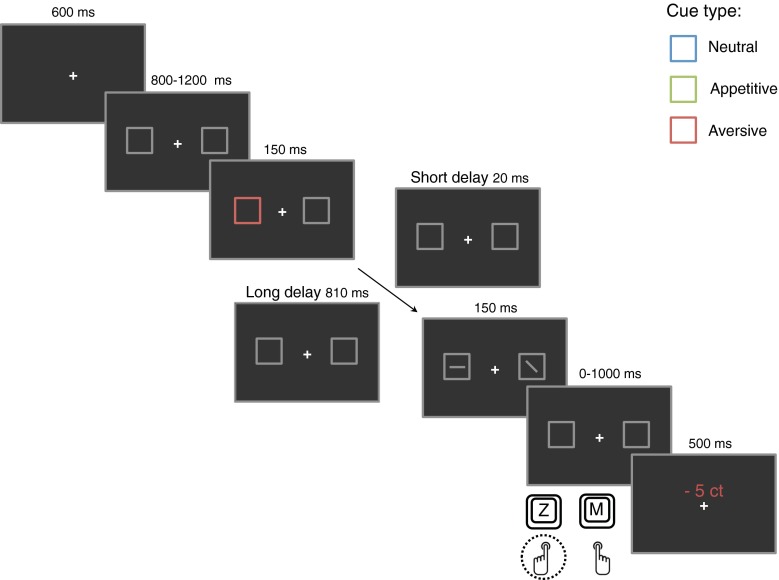
Schematic representation of the task. There was a blank 600 ms intertrial-interval. The trial started with a random 800-ms to 1,200-ms interval during which the target boxes were presented. A brief (150 ms) colored flash of the outline of one of the boxes served as the exogenous abrupt-onset cue. Half of the trials were followed by a short, 20-ms delay (above the arrow) and half by a long, 810-ms, delay (below the arrow). The target line (horizontal or vertical) and the distractor line (diagonal) were presented for 150 ms. Participants had a 1,000 ms response window to press the “Z” or “M” keyboard button. Feedback was presented for 500 ms. In the example, a valid aversive abrupt-onset cue trial is shown

All trials started with a random interval of 800 to 1,200 ms during which the two gray boxes were presented on the screen (see Fig. 1). Thereafter, the outline of one of the boxes was colored green, orange, or purple (the abrupt-onset cue) for 150 ms, followed by a 20-ms cue–target interval in short delay trials and a 810-ms cue–target interval in long delay trials. The target and distractor line were presented at the center of the two boxes for 150 ms. After target and distractor offset, there was a 1,000-ms response window in which participants had to press the “Z” keyboard button for horizontal and the “M” keyboard button for vertical targets. Reward feedback was presented for 500 ms immediately after the button press or after 1,000 ms when no response was made. All trials were separated by a blank intertrial-interval with a duration of 600 ms.

### Procedure

Participants were instructed to remain fixated at the central fixation cross at all times and respond as quickly and accurately as possible. Their task was to make a discrimination judgment between a horizontally or vertically oriented target line that appeared at the center of one of the boxes while a diagonal distractor line simultaneously appeared at the center of the other box. Participants were instructed that, prior to target appearance, one of the box outlines would briefly flash in a certain color but that this flash did not predict the target location (i.e., 50 % cue–target validity). Participants were NOT instructed about the specific color-reward contingencies. The appetitive, aversive, and neutral abrupt-onset cues were always followed by a monetary gain of 10 cents, a monetary loss of 5 cents, or no reward delivery, respectively, regardless of the participants’ response. Thus, even when no response, an incorrect response, or a too-slow response was given, the abrupt-onset cues were reinforced with their corresponding outcomes. In order to keep participants motivated to respond correctly, their overall accuracy needed to be above 75 % in order to receive the extra reward that could be earned. Furthermore, participants were instructed after the practice block that they had to respond faster than a certain individually adjusted reaction time threshold that was set to the average correct reaction time of the practice block.

Experiment 1 started with one practice block, followed by eight experimental blocks. Each block consisted of 48 trials, in which cue type (appetitive/aversive/neutral), delay (short/long), cue validity (valid/invalid), cue location (right/left), and target orientation (horizontal/vertical) were balanced and randomly presented. A valid trial was defined as the target being presented at the cued location, and an invalid trial was defined as the target being presented at the uncued (opposite to the cued) location. Because appetitive, aversive, and neutral cue trials were equally represented in each block and gains and losses were always administered, a fixed amount of monetary reward was earned during each block (€0.55). The extra reward over all blocks (€4.40) was paid to the participants if their overall accuracy was above 75 % correct. Including brakes between blocks, all participants were able to finish Experiment 1 within approximately 45 minutes.

### Statistical analyses

Accuracy was calculated as the percentage correct of all trials. Correct responses made within the 1,000-ms response window in the experimental blocks were included in the analyses. For both Experiments 1 and 2, we performed repeated-measures analyses of variance (ANOVA) on reaction time and accuracy, with cue type, delay, and cue validity as factors.

## Results

To investigate whether exogenous cueing could be modulated by value, we associated different-colored abrupt-onset cues with an appetitive, aversive, or neutral outcome. Mean reaction times for the different conditions of Experiment 1 are presented in Table 1. A repeated-measures ANOVA, with cue type (appetitive/aversive/neutral), delay (short/long), and cue validity (valid/invalid) as factors, revealed a significant main effect of delay, *F*(1, 29) = 67.861, *p* < .001, η_p_^2^ = .701, with faster reaction times in the long (483 ms) compared to the short (510 ms) delay, and a significant main effect of cue validity, *F*(1, 29) = 13.484, *p* < .001,η_p_^2^ = .317, with faster reaction times in validly cued (492 ms) compared to invalidly cued (500 ms) trials. There was no main effect of cue type, *F*(1, 29) < 1. Furthermore, there was a significant interaction between delay and cue validity, *F*(1, 29) = 31.314, *p* < .001,η_p_^2^ = .519, suggesting that cue validity had a different effect in the short delay compared to the long delay condition, a result that is related to the biphasic pattern of exogenous attentional deployment, typically observed in exogenous cueing tasks.

**Table 1:** Mean (standard deviation) reaction time and accuracy across participants in Experiment 1, separately presented for cue type, cue validity, and delay

	Appetitive cue		Aversive cue		Neutral cue	
	Valid	Invalid	Valid	Invalid	Valid	Invalid
Delay	Reaction time in ms					
Short	497 (71)	520 (72)	506 (60)	516 (72)	502 (67)	519 (75)
Long	477 (71)	485 (75)	488 (70)	479 (72)	484 (70)	481 (68)
	Accuracy (% correct)					
Short	91 (5)	87 (9)	89 (7)	89 (7)	91 (7)	89 (10)
Long	92 (6)	90 (7)	90 (8)	90 (9)	90 (8)	91 (7)

**Fig. 2:**
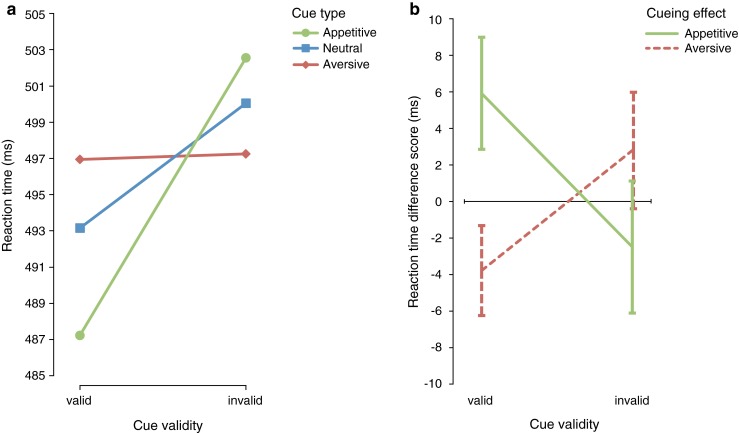
**a** The significant interaction between cue type and cue validity and (**b**) the reaction-time–difference scores (neutral minus appetitive and neutral minus aversive) show that compared to a neutral abrupt-onset cue, appetitive cues increase facilitation at the cued location whereas aversive cues decrease facilitation at the cued location. Error bars in this figure and following figures represent standard errors of the means

Crucially, there was a significant interaction between cue type and cue validity, *F*(2, 58) = 5.232, *p* = .008, η_p_^2^ = .153, suggesting that appetitive, aversive, and neutral exogenous cues differently modulated the reaction-time pattern for validly versus invalidly cued targets. Figure 2a displays this interaction and shows that, independent of delay, neutral abrupt-onset cues facilitate responding at the valid compared to the invalid location; appetitive abrupt-onset cues facilitate responding at the valid compared to the invalid location even more; and aversive cues show no facilitation for responding at the valid compared to the invalid location. In order to visualize the effect of associating the color of an abrupt-onset cue with an appetitive or aversive outcome compared to a pure (i.e., neutral) abrupt-onset cue, we calculated reaction-time–difference scores by taking the reaction times of the neutral cue type condition and separately subtracting the reaction times of the appetitive and aversive cue type condition from them. Figure 2b shows that compared to neutral abrupt-onset cues, associating an appetitive outcome with the color of an abrupt-onset cue increases the exogenous cueing effect and causes more attraction toward the location of the cue, whereas associating an aversive outcome with the color of an abrupt-onset cue decreases the exogenous cueing effect and causes reorienting away from the location of the cue.

**Fig. 3:**
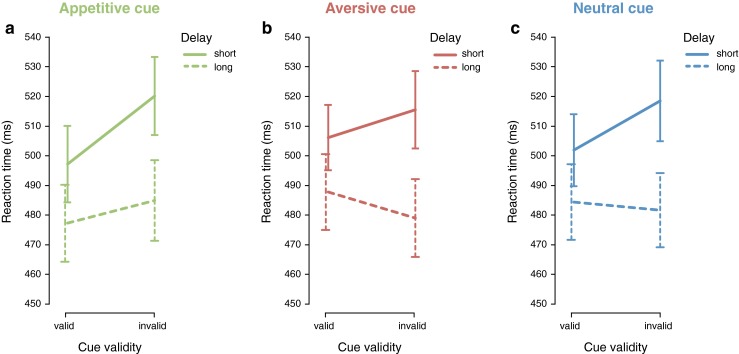
The interaction between delay and cue validity for (**a**) appetitive, (**b**) aversive, and (**c**) neutral abrupt-onset cues shows how orienting and reorienting processes are modulated by reward cue type. Appetitive cues show facilitation at the valid location in both the short and long delay, whereas aversive cues show facilitation in the short delay but inhibition of return in the long delay

While the three different cue types differ significantly in their overall validity effect, the three-way interaction between cue type, delay, and cue validity was not significant, *F*(1, 29) < 1. Therefore, according to prevalent statistical rules, the three-way interaction between cue type, delay, and cue validity should be disregarded. However, because cueing at short and long cue–target delays might refer to qualitatively different phenomena (i.e., cue facilitation in the short delay and IOR in the long delay; see Introduction), we kept the factor delay and explored the reaction time patterns for appetitive, aversive, and neutral cues separately at validly and invalidly cued locations for short and long delay trials. Figure 3 displays these interactions for the appetitive, aversive, and neutral cue-type conditions and reveals that all abrupt-onset cues facilitated responding at the location of the cue for short cue–target delay trials. Thus, as one can expect for short cue–target delays, all exogenous abrupt-onset cues produced a significant positive validity effect, although of different magnitude (see Table 1). For long cue–target delay trials, a mixture of results was observed with prolonged facilitation for appetitive cues, IOR for aversive cues, and no facilitation or IOR for neutral cues.

### Appetitive abrupt-onset cues

For appetitive abrupt-onset cues, a repeated-measures ANOVA on reaction time, with delay (short/long) and cue validity (valid/invalid) as factors, revealed a significant main effect of delay, *F*(1, 29) = 50.431, *p* < .001, η_p_^2^ = .635, with faster reaction times in the long (481 ms) compared to the short (509 ms) delay, a significant main effect of cue validity, *F*(1, 29) = 16.298, *p* < .001, η_p_^2^ = .360, with faster reaction times in valid (487 ms) compared to invalid (503 ms) trials, and a significant interaction between delay and cue validity, *F*(1, 29) = 6.772, *p* = .014, η_p_^2^ = .189. This interaction is shown in Fig. 3a and demonstrates that cues associated with a monetary win elicited significantly faster reaction times for valid (497 ms) compared to invalid (520 ms) cues in short delay trials, *t*(29) = 4.317, *p* < .001, and a trend for faster reaction times for valid (477 ms) compared to invalid (485 ms) cues in long delay trials, *t*(29) = 1.810, *p* = .081. This suggests that appetitive abrupt-onset cues elicited facilitation effects on initial orienting on short delay trials and that they provided prolonged facilitation at the validly cued location on long delay trials.

### Aversive abrupt-onset cues

For aversive abrupt-onset cues, a repeated-measures ANOVA on reaction time, with delay (short/long) and cue validity (valid/invalid) as factors, revealed a significant main effect of delay, *F*(1, 29) = 56.793, *p* < .001,η_p_^2^ = .662, with faster reaction times in the long (483 ms) compared to the short (511 ms) delay and a significant interaction between delay and validity, *F*(1, 29) = 12.493, *p* < .001, η_p_^2^ = .301. This interaction is shown in Fig. 3b and demonstrates that cues associated with a monetary loss elicited significantly faster reaction times for valid (506 ms) compared to invalid (516 ms) cues in short delay trials, *t*(29) = 2.049, *p* = .050, and significantly slower reaction times for valid (488 ms) compared to invalid (479 ms) cues in long delay trials, *t*(29) = 2.560, *p* = .016. This suggests that aversive abrupt-onset cues associated with a monetary loss elicited initial orienting, resulting in reaction-time facilitation at short delay trials, followed by reorienting attention away from the cued location, expressed by reaction-time facilitation at the uncued location at long delay trials.

### Neutral abrupt-onset cues

For neutral abrupt-onset cues, a repeated-measures ANOVA on reaction time, with delay (short/long) and cue validity (valid/invalid) as factors, revealed a significant main effect of delay, *F*(1, 29) = 50.500, *p* < .001,η_p_^2^ = .635, with faster reaction times in the long (483 ms) compared to the short (510 ms) delay and a significant interaction between delay and cue validity, *F*(1, 29) = 15.106, *p* < .001, η_p_^2^ = .342. This interaction is shown in Fig. 3c and demonstrates that neutral abrupt-onset cues elicited significantly faster reaction times for valid (502 ms) compared to invalid (518 ms) cues in short delay trials, *t*(29) = 3.699, *p* < .001, but no difference in response time for validly (484 ms) versus invalidly (mean = 482 ms) cued targets in long delay trials, *t*(29) < 1. This suggests that the neutral exogenous cues elicited initial orienting toward the cued location, resulting in reaction-time facilitation in short delay trials, yet there was no clear cueing effect on reaction time at long delay trials.

### Accuracy

A repeated-measures ANOVA on accuracy, with cue type (appetitive/aversive/neutral), delay (short/long), and cue validity (valid/invalid) as factors, revealed a significant main effect of cue validity *F*(1, 29) = 7.548, *p* = .010,η_p_^2^ = .207, with more accurate responses in validly (90.5 % correct) compared to invalidly (89.1 % correct) cued trials. Furthermore, there were no other significant main or interaction effects regarding accuracy. This suggests that the appetitive and aversive cue-type manipulations did not affect response accuracy and that the observed effects on reaction time are not due to speed–accuracy tradeoffs.

## Discussion

The results of Experiment 1 showed that appetitive cues strengthen attentional capture, whereas aversive cues reduce attentional capture compared to neutral cues and independent of cue–target delay. Exploring the attentional effects at the short and long cue–target delays separately, the data revealed that both appetitive and aversive cues initially facilitated responding at the validly cued location. However, at the long cue–target delays, this facilitation effect at the validly cued location remained present for gain-associated cues whereas it reversed for loss-associated cues. The fact that appetitive stimuli show initial and prolonged facilitation at their location might not be surprising because comparable results are observed in earlier work (Stankevich & Geng, 2015). Furthermore, it was also expected that the aversive and neutral abrupt-onset cues would initially capture attention because of their luminance onset. Surprisingly however, participants showed reorienting away from the validly cued location to the invalidly cued location (i.e., IOR) only in the long cue–target delay trials following aversive cues and not following neutral cues. As it might seem from Fig. 3 that there is not much difference between the attentional effects that aversive and neutral cues elicit, we conducted Experiment 2 to specifically investigate the time course of attention following aversive abrupt-onset cues.

## Experiment 2

To investigate the time course of attention following aversive and neutral abrupt-onset cues more in detail, we associated two different-colored exogenous cues with a neutral and aversive outcome in Experiment 2. To clearly distinguish between neutral and aversive cues, we used a blocked design in which blocks with a specifically colored neutral abrupt-onset cue (0 cents) and blocks with a different-colored aversive (-5 cents) abrupt-onset cue were alternatingly presented.

## Method

The overall methods used in Experiment 2 were highly similar to those used in Experiment 1, with a number of small changes.

### Participants

Twenty-five new participants (14 females, 18–34 years of age, mean = 23.7 years, standard deviation = 3.9 years) were recruited, who received €4.00 as a compensation for participation if their accuracy was below 75 % correct or €8.00 if their accuracy was above 75 % correct. All participants reported having normal or corrected-to-normal vision and gave written informed consent before participation. All research was approved by the Vrije Universiteit Faculty of Psychology ethics board and was conducted according to the principles of the Declaration of Helsinki. For counterbalancing reasons, one participant was replaced because his or her accuracy was below chance level in one or more conditions.

### Stimuli and apparatus

The stimuli and apparatus used in Experiment 2 were identical to those in Experiment 1. Because only aversive and neutral abrupt-onset cues were presented in Experiment 2, two out of the three colors (green/orange/purple) were coupled to an aversive and neutral outcome. The color–reward contingencies were counterbalanced across participants.

### Procedure

Experiment 2 started with one practice block in which neutral and aversive trials were presented intermixed. Then neutral and aversive blocks were alternatingly presented, for a total of 10 blocks. It was counterbalanced across participants whether they started with a neutral or aversive block. In Experiment 2, participants started with €16.00 and were told that the amount that was left at the end of the experiment would be paid if their accuracy was above 75 %. Each block consisted of 32 trials in which delay (short/long), cue validity (valid/invalid), cue location (right/left), and target orientation (horizontal/vertical) were balanced and randomly presented. Because aversive abrupt-onset cues were consistently followed by a loss of 5 cents and neutral abrupt-onset cues by no monetary win or loss, participants always lost €1.60 in aversive blocks and €0.00 in neutral blocks. The €8.00 that was left at the end of Experiment 2 was paid to the participants if their overall accuracy was above 75 % correct; otherwise, €4.00 was paid as a compensation for participation. Including breaks between blocks, all participants were able to finish Experiment 2 within approximately 35 minutes.

## Results

Mean reaction times for the different conditions of Experiment 2 are presented in Table 2. A repeated-measures ANOVA, with cue type (neutral/aversive), delay (short/long), and cue validity (valid/invalid) as factors, revealed a significant main effect of delay, *F*(1, 23) = 26.605, *p* < .001, η_p_^2^ = .536, with faster reaction times in the long (488 ms) compared to the short (506 ms) delay and a significant main effect of cue validity, *F*(1, 23) = 7.013, *p* = .014, η_p_^2^ = .234, with faster reaction times in validly cued (494 ms) compared to invalidly cued (500 ms) trials. There was no main effect of cue type, *F*(1, 23) < 1. Furthermore, there was a significant interaction between delay and cue validity, *F*(1, 23) = 8.533, *p* = .008, η_p_^2^ = .271, suggesting that cue validity had a different effect in the short compared to the long delay condition, an effect related to the biphasic pattern of exogenous attentional deployment. There was a trend for a significant interaction between cue type and cue validity, *F*(1, 23) = 3.868, *p* = .061, η_p_^2^ = .144, suggesting that attention was differently deployed to the validly and invalidly cued location following aversive and neutral cues. Crucially, the three-way interaction between cue type, delay, and cue validity was significant, *F*(1, 23) = 4.786, *p* = .039, η_p_^2^ = .172, suggesting that aversive and neutral abrupt-onset cues differently modulate the reaction-time patterns for validly and invalidly cued targets at short and long cue–target delays.

**Table 2:** Mean (standard deviation) reaction time and accuracy across participants in Experiment 2, separately presented for cue type, cue validity, and delay

	Neutral cue		Aversive cue	
	Valid	Invalid	Valid	Invalid
Delay	Reaction time in ms			
Short	496 (10)	512 (11)	501 (11)	515 (13)
Long	484 (12)	489 (11)	494 (13)	484 (12)
	Accuracy (% correct)			
Short	93 (6)	90 (7)	92 (5)	91 (5)
Long	93 (6)	93 (5)	93 (7)	94 (4)

**Fig. 4:**
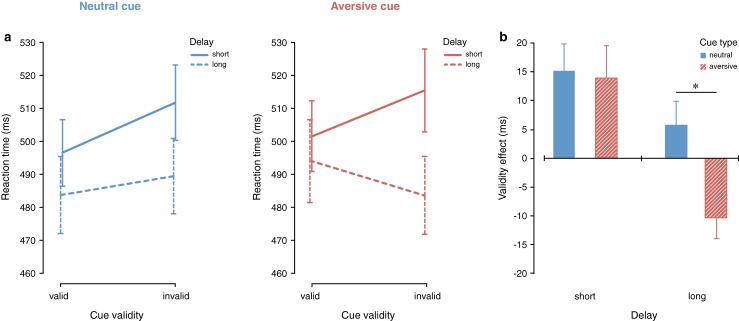
**a** The significant three-way interaction between cue type, delay, and cue validity shows that both neutral and aversive cues capture attention at the short delay. At the long delay, neutral cues show no validity effect, whereas aversive cues show inhibition of return. **b** The validity effect (invalid–valid) for neutral and aversive cues separately presented for short and long delays shows that aversive compared to neutral cues elicit significantly more reorienting away from the cued location when the cue target delay is long

The significant three-way interaction between cue type, delay, and cue validity is displayed in Fig. 4 and shows that attentional orienting and reorienting processes following aversive and neutral exogenous cues differ. At short cue–target delays, both neutral, *t*(23) = 3.234, *p* = .004, and aversive, *t*(23) = 2.455, *p* = .022, abrupt-onset cues elicit facilitation for responding at the validly compared to the invalidly cued location, with no significant difference in the validity effects between neutral and aversive abrupt-onset cues, *t*(23) > 1. However, at long cue–target delays, the neutral cue elicits no difference in responding at the validly compared to the invalidly cued location, *t*(23) = 1.36, whereas the aversive cue elicits significantly faster reaction times at the invalidly compared to the validly cued location, *t*(23) = 2.796, *p* = .010. The difference in response times at the validly and invalidly cued location for long cue–target delay trials differed significantly between neural and aversive abrupt-onset cues, *t*(23) = 2.461, *p* = .022 (see Fig. 4b). This suggests that aversive abrupt-onset cues initially capture attention similar to neutral abrupt-onset cues, but that with a longer cue–target delay aversive abrupt-onset cues elicit stronger reorienting away from the cued location compared to neutral abrupt-onset cues.

### Accuracy

A repeated-measures ANOVA on accuracy, with cue type (neutral/aversive), delay (short/long), and cue validity (valid/invalid) as factors, revealed a significant main effect of delay, *F*(1, 23) = 9.079, *p* = 0.006,η_p_^2^ = .283, with more accurate responses in long (93.4 % correct) compared to the short (91.4 % correct) delay trials, and a trend for a significant main effect of cue validity, *F*(1, 23) = 3.550, *p* = .072,η_p_^2^ = .134, with more accurate responses in validly (93.0 % correct) compared to invalidly (91.8 % correct) cued trials. Furthermore, there were no other significant main or interaction effects regarding accuracy. Because the response time and accuracy effects are consistent, this suggests that the observed effects on response time do not reflect a speed–accuracy trade-off.

## Discussion

The results of Experiment 2 showed that both neutral and aversive abrupt-onset cues initially facilitate responding at the cued location and that, at long cue–target delay trials, aversive cues elicited stronger reorienting away from the validly cued location toward the invalidly cued location (i.e., IOR) compared to neutral cues. These findings are congruent with the results from Experiment 1 and suggests that abrupt-onset cues associated with an aversive outcome elicit different attentional effects than neutral cues or cues associated with an appetitive outcome. Following an aversive abrupt-onset cue, attention is initially captured but later released and reoriented away from the location where the loss-associated cue was presented.

## General discussion

In the current study, we investigated whether orienting and reorienting processes following exogenous cueing could be modulated by associating abrupt-onset cues with appetitive or aversive outcomes. In two experiments, participants performed a Posner cueing task in which the color of the abrupt-onset cue signaled whether a gain, loss, or neutral (no gain or loss) outcome would follow. The results of Experiment 1 showed that there was no main effect of the type of exogenous cue but that the different abrupt-onset cues differently modulated the reaction-time pattern for validly compared to invalidly cued targets. Compared to neutral abrupt-onset cues and independently of cue–target delay, appetitive cues attracted attention more strongly toward the cued location, whereas aversive cues elicited reorienting away from the cued location. Exploring the time course following the different exogenous cues, we observed that at short cue–target delay trials, both the appetitive and aversive abrupt-onset cue facilitated performance at the validly cued location, whereas, at long cue–target delay trials, this facilitation effect remained present for cues associated with a monetary win while it reversed for cues associated with a monetary loss. These findings suggest that, initially, both appetitive and aversive cues capture attention. Yet, if time passes, attention remains lingering at the cued location for appetitive cues, whereas attention is reoriented away from the location of aversive cues. In Experiment 2, we specifically investigated the development of attentional deployment over time following exogenous cueing with aversive abrupt-onset cues and confirmed the findings from Experiment 1. The results showed that aversive cues, indeed, captured attention and elicited facilitation at the cued location initially, and afterward attention was reoriented toward the uncued location. Crucially, when the cue–target delay was long, reorienting away from the location of the abrupt-onset cue was significantly stronger following the presentation of aversive compared to neutral cues. Together these findings indicate that the attentional effects that abrupt-onset cues elicit can be differently modulated by associating them with appetitive and aversive outcomes.

The present results reveal differences in attentional deployment after cueing with abrupt-onset cues that are associated with a monetary win and monetary loss, which suggests that value-driven attentional biases are sensitive to differences in valence. Because Tversky and Kahneman’s loss-aversion theory states that value slopes for losses are steeper than those for gains (Tversky & Kahneman, 1991), we chose for an asymmetrical design (i.e., a gain of 10 cents and a loss of 5 cents) in an attempt to keep the subjective value of both the appetitive and aversive cues equivalent. Consequentially, a value-driven mechanism, insensitive to valence differences, should have produced similar attentional biases following the appetitive and aversive abrupt-onset cues. However, compared to neutral cues we observed more attraction toward the location of the gain-associated cue in Experiment 1 and more reorienting away from the loss-associated cues in Experiments 1 and 2. This suggests that cues associated with an appetitive outcome can strengthen attentional capture and orienting processes, whereas cues associated with an aversive outcome reduce attentional capture and promote reorienting. We like to emphasize that in the present study, the cues associated with an aversive outcome were always followed by an actual loss that could not be avoided. This is different from a condition in which a monetary loss can be prevented by giving a fast and correct response. For example, in a recent study by Wentura and colleagues (Wentura, Müller, & Rothermund, 2014), two colors were associated with gains and losses and two colors were associated with neutral outcomes in a training task. Participants gained money if the reward-associated color was present and their response was fast and correct, and lost money if the punishment-associated color was present and their response was too slow or incorrect. After the training phase, participants performed the additional singleton task, during which the additional singleton was presented in the reward-associated, punishment-associated, or one of the neutral colors. Reaction times were slower for both the reward and punishment condition compared to the neutral condition, with no difference in reaction time between the reward and punishment condition. Therefore, the authors argued that the general relevance of the reward- and punishment-associated stimuli caused a similar attentional effect. As there is no cue–target delay factor using the additional singleton paradigm, the results are comparable with the fact that both reward and punishment cues initially captured attention in Experiment 1. Yet, one could argue that the underlying mechanisms are different, as if, in essence, avoiding a monetary loss or obtaining a monetary win is the same because both increase the incentive to give a correct and fast response in order to end up with the highest monetary outcome. Consequentially, both conditions in the study by Wentura and colleagues most likely recruited similar endogenous motivational processes that resulted in the same attentional bias (i.e., faster reaction times and/or improved accuracy). However, in our study there was no relationship between the participants’ response and the appetitive and aversive outcome such that monetary gains and losses could never be avoided. Thus, a correct and fast response always led to a (positively valenced) win when the appetitive cue was presented but to a (negatively valenced) loss when the aversive cue was presented. This is in contrast to the study of Wentura and colleagues, in which winning or losing money was dependent on the participants’ response. Given that monetary gains and losses were inevitable in the present study, it is not surprising that the appetitive and aversive exogenous cues elicited different, valence-specific, value-driven attentional biases.

As mentioned, one of the strengths of the current study was that the abrupt-onset cues were consistently followed by their corresponding outcome association, such that the specifically colored cues always signaled whether a monetary win, loss, or no win or loss would be administered. The outcome of a trial was independent of the participants’ response and performance, but to keep participants engaged in the task, we instructed them that their overall accuracy needed to be above 75 % in order to receive the extra monetary rewards that were earned during Experiment 1 or not lost during Experiment 2. Because participants had no influence on winning or losing money on a trial-by-trial basis, their only incentive should have been to be as fast and accurate as possible to score 75 % correct or above. Therefore, we believe that the observed attentional biases for appetitive and aversive abrupt-onset cues are not due to differential motivational processing. This is supported by the lack of a main effect of exogenous cue type on reaction time and accuracy in both Experiments 1 and 2, which indicates that perceiving the appetitive or aversive cue did not elicit general, non-spatially specific, performance benefits or costs. Even in Experiment 2, where there was possibly more room for motivational processing to modulate attentional effects due to the blocked design, there was no general effect of cue type. This means that overall performance was not influenced by the fact that participants were losing money in some blocks and did not lose any money in other blocks. Possibly, the absence of this effect was due to the alternating presentation and the short length (32 trials) of the individual blocks. If motivational process related to losing money operate on a larger timescale, then behavioral effects would be revealed only when longer blocks were used. Therefore we like to argue that by using the current design, we believe that the observed attentional biases do not just reflect voluntary motivational processing. This is in line with an earlier study, showing that reward-induced motivational processes enhance IOR (Bucker & Theeuwes, 2014) instead of diminishing it. In that study, the same exogenous cue was presented throughout the whole experiment, and reward was manipulated in a blocked-wise manner with high- and low-reward outcome blocks. The results showed typical cue facilitation in the short cue–target delay trials under both high- and low-motivational conditions, whereas IOR was observed only in the long cue–target delay trials when participants were highly motivated. Therefore, it was concluded that reward-induced motivation had a clear endogenous effect on reorienting and inhibitory processes when motivation was high. However when the outcome of a trial is directly coupled to the specific color of the abrupt-onset cue, as in the present study, opposing results are observed, with no IOR but rather with prolonged facilitation, following cues that signal a monetary reward.

With regard to orienting and inhibitory processes, the present results reveal that appetitive abrupt-onset cues elicited quick and prolonged facilitation for responding at the validly compared to the invalidly cued location. This implies that reward-associated exogenous cues immediately evoked a strong attraction toward their location followed by sustained attention at that location for at least up to a second. However, because the current design cannot disentangle sustained attention from delayed reorienting, an alternative explanation might be that reorienting processes are recruited to a lesser extent when a gain-associated cue is presented. Following this reasoning, it could be that the turning point from cue facilitation to IOR is delayed for cues that are associated with a monetary win. Similar results have been observed in another study examining reward-selection history effects at various cue–target delays (0, 200, 400, or 800 ms; Stankevich & Geng, 2015). However, in that study there was no exogenous capture, because the reward and no-reward associated stimulus were presented simultaneously. Participants performed a discrimination judgment on the target that appeared within one of the stimuli, and for correct responses participants received either a reward or nothing, depending on the color of the circle that contained the target. The results showed that targets in the reward circle enjoyed attentional priority over those in the no-reward circle, and this effect did not change as a function of time. The authors concluded that the reward-associated cue seemed to provide fast, exogenous-like facilitation for target discrimination performance at the shortest cue–target delays as well as sustained performance benefits for the longer cue–targets delays. This temporal profile is similar to the temporal profile we observed for the appetitive abrupt-onset cues in the current study, with fast (20-ms cue–target delay) and sustained (810-ms cue–target delay) orienting toward the validly cued location and a lack of IOR. An important difference between the two studies is that the reward information in Stankevich and Geng (2015) was presented throughout the whole trial (even when the target was presented) and that attending the high-reward associated stimulus was beneficial for reward payout (although the target location was not predicted by the reward-associated stimulus, reward could only be obtained for targets appearing in the reward-associated stimulus). Therefore, the sustained reward effect might have been observed because orienting toward the reward-associated stimulus was always beneficial for that trials reward payout. It is likely that initial value-driven attentional capture was followed by voluntary, controlled guidance of attention toward the reward-signaling stimulus at longer cue–target delays in Stankevich and Geng (2015) because observers had an incentive to do so. On the contrary, in the present study, the reward-associated abrupt-onset cue was presented for only 150 ms and never presented simultaneously with the target stimulus. Furthermore, there was no incentive for observers to voluntarily maintain their attention at the location of the reward-associated exogenous cue because the rewarding outcome was location and response independent. However, despite the lack of a direct top-down incentive to maintain attention at the location of the reward-associated cue, one might argue that this sustained orienting (or lack of reorienting) effect reflects the deployment of endogenous attention. After being captured by the reward-associated cue, top-down processes take over to maintain attention at the positively associated location, which causes attention to dwell at the cued location or prevents attention to reorient to the uncued location.

In contrast with appetitive, win-associated abrupt-onset cues, the results of Experiments 1 and 2 together suggest that the aversive, loss-associated abrupt-onset cues quickly captured attention, after which attention was reoriented from the cued location to the uncued location. Immediate orienting toward the loss-associated abrupt-onset cues is in line with research showing that a stimulus associated with an electric shock, which is also considered to be aversive, has the ability to capture attention (Schmidt et al., 2015) when presented for 100 ms simultaneously with an equally salient stimulus that is not associated with an electric shock. Crucially, however, in a subsequent study, Schmidt, Belopolsky, and Theeuwes (2016) showed that after attention was captured by the stimulus associated with an electric shock, attention remained at that location, even for long cue–target delays. This is unlike the present results, which show that initial capture by the aversive stimulus is followed by inhibition, exposed by faster reaction times at the uncued compared to the cued location for long cue–target delays. It is likely that these differences are because of the differences in the nature of the aversive events (i.e., losing money vs. receiving an electric shock) and the instrumental relation that the electric shock had in the study by Schmidt and colleagues (i.e., electric shocks were only delivered when participants responded slower than a certain threshold). Here, however, the aversive stimulus was associated with and always followed by a monetary loss, independent of the participants’ response. In the study by Schmidt and colleagues, where the aversive and potentially threatening electric shock could be avoided by showing the appropriate behavior, observers had a very good incentive to maintain attention at the location of the stimulus that was coupled to the electric shock. However, in the present study, there was no incentive to maintain attention at the location of the aversive exogenous cue that inevitably signaled a monetary loss, once it was detected. Similar to the suggestion that endogenous processes maintained attention at the location of appetitive cues following initial capture, it is likely that, after initial capture by aversive cues, top-down processes reoriented attention away from the loss-associated location. Following the same reasoning, it can be explained that no significant IOR was observed following neutral cues in both experiments. That is, in a context with aversive, loss-associated cues, a neutral cue can be considered to be “safe” or even positive. Consequently, top-down processes might have maintained attention at the location of the neutral cue to some extent or prevented reorienting processes that would have normally led to IOR.

To summarize, Experiment 1 reveals that compared to neutral abrupt-onset cues and independent of cue–target delay, appetitive abrupt-onset cues attract attention toward their location, whereas aversive abrupt-onset cues elicit reorienting away from their location. Exploratory analyses regarding the time course of attention following the different exogenous cues show that all abrupt-onset cues initially capture attention regardless if they are associated with a win, loss, or neutral outcome. Following initial capture, attention is maintained at the location of appetitive abrupt-onset cues, whereas attention is released and reoriented away from the location of aversive abrupt-onset cues. This latter finding was replicated and confirmed by the results of Experiment 2. Although associations with monetary win or loss seem to have little effect on initial exogenous capture by abrupt-onset cues, they greatly influence the deployment of attention later in time, in a valence-specific manner. Together, these findings suggest that the attentional effects that abrupt-onset cues elicit can be differently modulated by associating them with appetitive and aversive outcomes.
